# Airway Epithelial Cells Differentially Adapt Their Iron Metabolism to Infection With *Klebsiella pneumoniae* and *Escherichia coli In Vitro*

**DOI:** 10.3389/fcimb.2022.875543

**Published:** 2022-05-18

**Authors:** Philipp Grubwieser, Alexander Hoffmann, Richard Hilbe, Markus Seifert, Thomas Sonnweber, Nina Böck, Igor Theurl, Günter Weiss, Manfred Nairz

**Affiliations:** ^1^ Department of Internal Medicine II, Infectious Diseases, Immunology, Rheumatology, Medical University of Innsbruck, Innsbruck, Austria; ^2^ Christian Doppler Laboratory for Iron Metabolism and Anemia Research, Medical University of Innsbruck, Innsbruck, Austria; ^3^ Biocenter, Institute of Bioinformatics, Medical University of Innsbruck, Innsbruck, Austria

**Keywords:** nutritional immunity, iron, ferroportin, airway epithelium, nosocomial pneumonia, *E. coli*, *K. pneumoniae*

## Abstract

**Background:**

Pneumonia is often elicited by bacteria and can be associated with a severe clinical course, respiratory failure and the need for mechanical ventilation. In the alveolus, type-2-alveolar-epithelial-cells (AECII) contribute to innate immune functions. We hypothesized that AECII actively adapt cellular iron homeostasis to restrict this essential nutrient from invading pathogens – a defense strategy termed ‘nutritional immunity’, hitherto mainly demonstrated for myeloid cells.

**Methods:**

We established an *in-vitro* infection model using the human AECII-like cell line A549. We infected cells with *Klebsiella pneumoniae* (*K. pneumoniae*) and *Escherichia coli* (*E. coli*), two gram-negative bacteria with different modes of infection and frequent causes of hospital-acquired pneumonia. We followed the entry and intracellular growth of these gram-negative bacteria and analyzed differential gene expression and protein levels of key inflammatory and iron metabolism molecules.

**Results:**

Both, *K. pneumoniae* and *E. coli* are able to invade A549 cells, whereas only *K. pneumoniae* is capable of proliferating intracellularly. After peak bacterial burden, the number of intracellular pathogens declines, suggesting that epithelial cells initiate antimicrobial immune effector pathways to combat bacterial proliferation. The extracellular pathogen *E. coli* induces an iron retention phenotype in A549 cells, mainly characterized by the downregulation of the pivotal iron exporter ferroportin, the upregulation of the iron importer transferrin-receptor-1 and corresponding induction of the iron storage protein ferritin. In contrast, cells infected with the facultative intracellular bacterium *K. pneumoniae* exhibit an iron export phenotype indicated by ferroportin upregulation. This differential regulation of iron homeostasis and the pathogen-specific inflammatory reaction is likely mediated by oxidative stress.

**Conclusion:**

AECII-derived A549 cells show pathogen-specific innate immune functions and adapt their iron handling in response to infection. The differential regulation of iron transporters depends on the preferential intra- or extracellular localization of the pathogen and likely aims at limiting bacterial iron availability.

## Introduction

Infections of the lower respiratory tract, including bacterial pneumonia, remain a major public health problem and a leading cause of death worldwide (Mizgerd, 2008; GBD Collaborators, L. R. I., 2018) . A large variety of microorganisms, including bacteria, viruses and fungi, can invade the distal airways and alveoli, causing a pronounced acute inflammatory response in the lung parenchyma and thus the clinical syndrome of pneumonia (Torres et al., 2021). Based on its etiology, pneumonia is broadly categorized into community-acquired pneumonia (CAP) and hospital-acquired pneumonia (HAP). The latter form is the second most common cause of hospital-acquired infection (Haque et al., 2018). Ventilation-associated pneumonia (VAP), a subset of HAP, affects 10-25% of all patients on mechanical ventilation and is the most common cause of nosocomial infection and death in the ICU (Torres et al., 2017; Torres et al., 2021).

In each entity, the most prevalent causative microorganisms differ. CAP is commonly caused by *Streptococcus pneumoniae*, *Haemophilus influenzae* or respiratory viruses. In contrast, HAP is predominantly elicited by *Staphylococcus aureus* or by different gram-negative bacilli such as *Klebsiella pneumoniae, Pseudomonas aeruginosa* and *Escherichia coli* (Jean et al., 2020).

The clinical outcome of pneumonia is tightly linked to the virulence of the invading pathogen and the inflammatory response in the lung. The latter requires a balancing act between clearance of the causative agent and minimizing tissue damage resulting from the host response (Quinton et al., 2018). Several host defense systems work in tandem to achieve this, with the innate immune system playing a crucial role in detecting and inhibiting initial bacterial proliferation, thus containing the infection (Bals and Hiemstra, 2004; Weitnauer et al., 2016). As the first line of defense against invading pathogens, epithelial cells are increasingly recognized to directly contribute to innate immune functions. Specifically in the alveolus, type-II alveolar epithelial cells (AECII) have been shown to play critical roles in host defense: pathogen detection, intercellular communication and production of bactericidal compounds (Bals and Hiemstra, 2004; Chuquimia et al., 2013). Several *in vitro* and *in vivo* studies conducted in mice have revealed, that AECII internalize bacterial pathogens, including *K. pneumoniae* (de Astorza et al., 2004; Chuquimia et al., 2012; Hsu et al., 2015).

One decisive factor in host-pathogen interactions is the combat for nutrients essential for both opponents. The sequestration of these nutrients such as the trace metal iron from pathogens is regarded as an efficient host defense strategy, a concept known as *nutritional immunity* (Núñez et al., 2018; Nairz and Weiss, 2020). Iron is essential to almost all forms of life, contingent on its ability to act as a universal redox catalyst and involvement as a co-factor in an abundance of biochemical processes critical to life (Núñez et al., 2018). Proliferation capacity of bacteria is affected by the amount of iron in their environment (Griffiths, 1991). In the intestine, increased dietary iron may even drive the selection of microorganisms to commensalism, emphasizing this trace metals multifaceted role in host pathogen interactions (Sanchez et al., 2018). In the case of infection, the host has evolved several mechanisms to take advantage of bacterial iron demand by adapting the spatio-temporal regulation of its iron metabolism. Dependent on the extra- or intracellular localization of a pathogen, the host response withholds iron from the pathogen’s compartment, thus limiting bacterial proliferation (Nairz et al., 2010; Haschka et al., 2021a). In parallel, bacteria have developed diverse mechanisms to counteract *nutritional immunity* and acquire iron to sustain survival and proliferation, once confined in the microenvironment of the host. Exemplary of this co-evolutionary arms race are high-affinity iron-binding molecules known as siderophores (eg. *enterobactin*), secreted predominantly by gram-negative bacterial species to scavenge iron from their host (Behnsen and Raffatellu, 2016; Kramer et al., 2020). In turn, several host cells, including the airway epithelium, produce the immune mediator lipocalin-2 (Neutrophil-gelatinase associated lipocalin, NGAL), which binds iron-loaded *enterobactin*, rendering it inaccessible for bacterial uptake. Some *Enterobacteriaecae* (e.g. *K. pneumoniae)* are able to evade this immune strategy by producing alternative siderophores that NGAL cannot bind, such as yersiniabactin and salmochelin. In the case of *K. pneumoniae*, the expression of yersiniabactin is a virulence factor promoting respiratory tract infections through evasion of NGAL (Bachman et al., 2011). Systemically, iron overload is associated with an increased risk for infections with multiple bacterial pathogens, including *E. coli* (Parrow et al., 2013).

In the lung, sequestration of iron and other trace metals affects immune cell function and alters the response to infection, rendering tight regulation of iron bioavailability in the respiratory system vital to the host (Healy et al., 2021). In airway epithelia, typical iron transport proteins have been identified, including the cellular iron influx transporter transferrin-receptor-1 (TFR1) (Heilig et al., 2006), the cellular iron exporter ferroportin (FPN) (Yang et al., 2005), and the iron storage protein ferritin (FT) (Ghio et al., 1998). The ability to differentially regulate cellular iron metabolism in response to infection with intra- or extracellular proliferating bacteria greatly benefits host defense, and has thus far mostly been ascribed to myeloid cells (Soares and Weiss, 2015).

In times of increasing antibiotic resistance, alternative strategies to combat bacterial infections are in need. Thus, the concept of *nutritional immunity* remains a highly relevant and evolving field. This study aimed to examine the reaction of AECII-derived A549 cells to infection with *K. pneumoniae* and *E. coli*, both of which can cause HAP, one of the most severe and difficult-to-treat forms of respiratory tract infections. We adapted an *in vitro* model to investigate the differential regulation of iron homeostasis and inflammatory reaction induced by these gram-negative pathogens.

## Material and Methods

### Cell and Bacterial Culture

We used the human cell line A549 (DMSZ, ACC 107), which closely recapitulates the AECII phenotype as our model system (Nardone and Andrews, 1979; Foster et al., 1998). Cells were propagated in DMEM (PAN-Biotech) containing 10% FBS (PAN-Biotech) and 1% Penicillin/Streptomycin (Lonza). For infection experiments, cells were washed with PBS and seeded into 6-well plates (Greiner bio-one) or 10cm dishes (Falcon) in an antibiotic-free medium containing 1% FBS.

Bacteria (*E. coli* ATCC 25922 and *K. pneumoniae* ATCC 43816) were grown from overnight cultures under sterile conditions in LB Broth (Sigma-Aldrich) to late logarithmic phase [optical density 600nm (OD600) 0.45-0.6]. Bacterial counts were determined before each experiment using a cell counter and analyzer (CASY, 45µm capillary, OLS OMNI Life Science).

For growth assays, bacteria were diluted to OD_600_ of 0.005 in cell culture medium in 96-well plates (Greiner bio-one), and directly afterwards incubated in an automated microplate reader (Spark, TECAN) at 37°C, 5% CO2 under constant double orbital shaking. OD_600_ was measured every 5min for a total of 10h.

For heat-inactivation, bacteria were incubated at 70°C for 20min and afterwards plated on LB plates to confirm absence of viable bacteria.

### Fluorescence Microscopy

Bacteria were made electro-competent using glycerol/mannitol density step centrifugation, as described in an established protocol (Warren, 2011). The plasmid pBC20 with a gene encoding for the fluorescent protein Ypet (517/530nm) downstream of the constitutively active *PybaJ* promoter was electroporated into *E. coli* and *K. pneumoniae*. To visualize possible cell invasion of bacteria, A549 cells were seeded onto sterilized coverslips inside 6-well plates and infected with fluorescent bacteria at a multiplicity of infection (MOI) of 10 for 2h. Subsequently, cells on coverslips were washed thrice with PBS (Lonza) and fixed with 4% paraformaldehyde solution for 20min. Samples were permeabilized with 0.2% Triton X-100 (Roth) for 30min. Alexa-flour-594-labeled phalloidin (Invitrogen, A12381) was used to stain the actin cytoskeleton, and 4′,6-diamidino-2-phenylindole (DAPI, BioLegend, 422801) was used to stain nuclei for 30 min at room temperature. Samples were mounted with Faramount Mounting Medium (Dako, S3025) onto slides. Imaging was performed immediately after sample preparation using a VS120-S6 fluorescence microscope (Olympus). Images were captured with a 40-x objective using 387/440nm (DAPI), 485/525nm (Ypet), and 560/607nm (phalloidin) lasers and filters.

### Gentamicin Protection Assay

We applied a gentamicin-protection assay (Elsinghorst, 1994) to establish the entry and presence of viable (colony-forming) intracellular bacteria over a period of up to 24h with the following adaptions: cells were infected with either *E. coli* or *K. pneumoniae* at a MOI of 10. After a 2h incubation phase, cells were washed thrice with PBS containing gentamicin (Life Technologies, 50µg/ml) and incubated in fresh medium containing 1% FCS and gentamicin (25µg/ml) during the intracellular infection phase. Gentamicin treatment during the intracellular infection phase facilitates the killing of extracellular bacteria, but does not affect pathogens that have entered cells, as gentamicin has no intracellular bactericidal activity (Elsinghorst, 1994). In parallel, we subjected uninfected control cells to identical steps of washing and incubation with gentamicin. At indicated time points (0h = directly after incubation phase), cells were washed thrice again in PBS and lysed in 0.5% sodium deoxycholic acid (Sigma-Aldrich). Cell lysates were plated immediately on LB plates, and colony-forming units (CFUs) were quantified after overnight incubation. A timeline of experimental procedures is depicted in **Supplementary Figure 1**.

Where indicated, cells were stimulated 3h before the incubation phase and during the intracellular infection phase with 25µM iron (III) nitrate nonahydrate (Sigma-Aldrich). Where appropriate, cells were treated with 5mM N-acetyl-cysteine (NAC) 20min before infection, during incubation phase and intracellular infection phase. When iron-loaded cells were infected, bacteria were iron starved in an iron-free medium (IMDM, Lonza) while growing to the late logarithmic phase.

### Quantitative Real-Time PCR

The quantitative real-time PCR was carried out as described elsewhere (Hoffmann et al., 2021). In brief, total RNA isolation was prepared using acid guanidinium thiocyanate-phenol-chloroform extraction with peqGOLD Tri-Fast™ (Peqlab). For reverse transcription 2 µg RNA, random hexamer primers (200 ng/µl) (Roche), dNTPs (10 mM) (GE Healthcare LifeSciences) 20 U RNasin (Promega) and 200 U M-MLV reverse transcriptase (Invitrogen) in first-strand buffer (Invitrogen) were used. Ssofast Probes Supermix and Ssofast EvaGreen Supermix (Bio-Rad Laboratories GmbH) were used according to the manufacturer’s instructions. Real-time PCR reactions were performed on QuantStudio 3 and 5 real-time PCR systems (Thermo Fisher Scientific). Gene expression was normalized using the ΔΔct method using *Tubulin* (*TUB*) and *ornithine decarboxylase antizyme 1* (*OAZ1)* as reference transcripts.

The following TaqMan PCR primers and probes were used (all 5’→3’; primer forward; primer reverse; probe):

*TUB*: TCCTTCAACACCTTCTTCAGTGAGACG; GGTGCCAGTGCGAACTTCATCA; ATGTGCCCCGGGCAGTGTTTGTAGACTTG

*OAZ1*: GGATCCTCAATAGCCACTGC; TACAGCAGTGGAGGGAGACC; TGGATGGTGGCGCTGGGTTTATC

*FPN*: TGACCAGGGCGGGAGA; GAGGTCAGGTAGTCGGCCAA; CACAACCGCCAGAGAGGATGCTGTG

*TFR1*: TCCCAGCAGTTTCTTTCTGTTTT; CTCAATCAGTTCCTTATAGGTGTCCA; CGAGGACACAGATTATCCTTATTTGGGTACCACC

*HAMP*: AGACGGGACAACTTGCAG, TCCCACACTTTGATCGATGAC, ACACCCACTTCCCCATCTGCATT

*IL-1B*: CTG CTC TGG GAT TCT CTT CAG; ATC TGT TTA GGG CCA TCA GC

*IL-6*: AGCCCACCGGGAACGAAAGAGA; AAGGCAGCAGGCAACACCAGG; AACTCCTTCTCCACAAGCGCCTTC

*IL-8*: AGCCTTCCTGATTTCTGCAG; GTCCACTCTCAATCACTCTCA

*HO1*: TCAGGCAGAGGGTGATAGAAG; TTGGTGTCATGGGTCAGC; TGGATGTTGAGCAGGAACGCAGT

*FTH*: CTCCTACGTTTACCTGTCCATG; TTTCTCAGCATGTTCCCTCTC

*FTL*: AACCATGAGCTCCCAGATTC; CGGTCGAAATAGAAGCCCAG

*KEAP1*: AACAGAGACGTGGACTTTCG; GTGTCTGTATCTGGGTCGTAAC

### Western Blot

Protein extraction and Western blotting were performed as described previously (Hoffmann et al., 2021). The following antibodies were used: a rabbit FPN antibody [1:2000; Eurogentec, custom made (Petzer et al., 2020)], a mouse TFR1 antibody (1:1000; Sigma Cat# SAB4300398), a rabbit FT antibody (1:500; Sigma), a rabbit NGAL antibody (1:1000 Abcam, ab63929), a rabbit NRF2 antibody (1:1000, Abcam, ab31163), and a rabbit actin antibody (1:500; Sigma Cat# A2066). Appropriate HRP-conjugated secondary antibodies (1:2000, anti-rabbit; Dako Cat# P0399 1:4000, anti-mouse; Dako Cat# P0447) were used. For quantification, densitometry data were acquired on a ChemiDoc Touch Imaging System (Bio-Rad) and analyzed with Image Lab 5.2.1. (Bio-Rad Laboratories GmbH).

### ROS Assay

For evaluation of ROS in A549 cells during the 2h incubation phase, A549 cells were seeded into 12-well-plates in antibiotic-free medium. Cells were infected with either *E. coli* or *K. pneumoniae* at MOI of 10 and stained with 2.5µM CellROX Deep Red Reagent (Thermo Fisher Scientific). Immediately afterwards, infected cells were incubated at 37°C in an automated multimode microplate reader (Spark, TECAN) and 670nm fluorescence was read at 16 different localizations in each well every 5min for a total of 2h

### Statistical Analysis

Statistical analysis was carried out using GraphPad Prism version 9.1 for Windows and Mac (GraphPad Software). Data are presented as mean with 95% CI or SEM as dispersion characteristic. Significant differences between groups were determined using ANOVA with *post-hoc* analysis. Multiple comparisons were adjusted using Tukey’s or Holm-Šídák’s methods. For non-normal distributed data, as evaluated by Kolmogorov-Smirnov- or Shapiro-Wilk-test, Kruskal-Wallis test with Dunn’s multiple comparisons test was performed. p < 0.05 was used as the significance threshold.

## Results

### *E. coli* and *K. pneumoniae* Infect Alveolar Epithelial Cells

**Figure 1:**
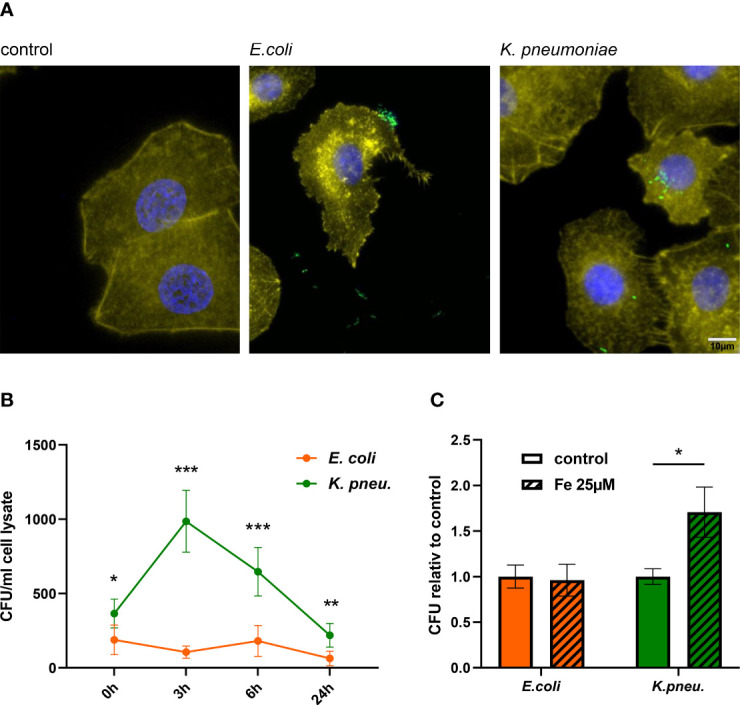
Immune fluorescence imaging reveals the predominant localization of bacteria: *E. coli* in the extracellular space and *K. pneumoniae* in the intracellular space of A549 cells **(A)**. Representative images, showing Ypet expressing bacteria (green) infecting A549 cells (DAPI= blue, phalloidin= yellow) at 400x magnification with a 10µm scale bar. Time course of bacterial load (recovered intracellular CFU) in A549 cells **(B)**. A549 cells were infected with *E. coli* or *K. pneumoniae* for 2h at MOI of 10 and were lysed directly after incubation phase (0h time point) or at noted time intervals of intracellular infection. Lysates were plated onto LB-agar plates for CFU quantification. Data are shown as mean CFU/ml lysate ± 95% CI of three separate experiments.Intracellular bacterial load increases in *K. pneumoniae* infected, iron-loaded cells **(C)**.A549 cells were stimulated with 25µM iron (III) nitrate nonahydrate 3h before infection. Cells were infected with either *E. coli* or *K. pneumoniae* at MOI of 10 for 2h in fresh iron-adequate medium. Subsequently, cells were washed thoroughly and further incubated in gentamicin-containing medium ± 25µM iron(III) nitrate nonahydrate for 6h, until cells were lysed, and intracellular bacteria plated for CFU quantification. Data from three separate experiments are shown as mean ± SEM, normalized to the corresponding control condition.* denotes p<0.05, ** denotes p<0.01, *** denotes p<0.001 for *post-hoc* statistical testing. CFU, colony-forming-units*; K. pneu*., *K*. *pneumonia*.

We infected A549 cells with either of the two pathogens under the same conditions. Applying fluorescence microscopy using transformed bacteria that constitutively express Ypet fluorescent protein, we aimed to shed light on the cellular localization of the pathogens upon *in vitro* infection. As depicted in **Figure 1A**, imaging revealed bacteria predominantly in the extracellular space in the case of *E. coli* infection. In contrast, large numbers of *K. pneumoniae* were found intracellularly. Additional, lower magnification microscopy images are provided in **Supplementary Figure 2**.

Using an adapted gentamicin protection assay, we saw that both bacterial pathogens, *E. coli* and *K. pneumoniae* are capable of invading A549 cells, with significantly more *K. pneumoniae* entering cells directly after the incubation phase (**Figure 1B** and **Supplementary Figure 1**). After invasion, only *K. pneumoniae* was capable of intracellular proliferation, with a maximum of viable intracellular bacteria being recovered after 3h of intracellular infection. After this peak bacterial load, numbers of recovered *K. pneumoniae* decreased, suggesting that epithelial cells initiate antimicrobial immune pathways to effectively combat intracellular bacterial proliferation and decrease bacterial numbers. *E. coli*, regarded as a typical extracellular pathogen (Kaper et al., 2004), was unable to sustain intracellular proliferation in our experiments, indicated by an invariable and low bacterial load at all time intervals.

To examine the impact of elevated intracellular iron levels on bacterial growth capacity, we stimulated A549 cells with 25µM iron(III)-nitrate prior to infection and further during intracellular infection phase. **Figure 1C** demonstrates that cells loaded with iron exhibited higher bacterial burden, when infected with *K. pneumoniae*. In contrast, the extracellular pathogen *E. coli* showed no increase in intracellular proliferation. This indicates, that while higher intracellular iron concentration in epithelial cells promote the growth of *K. pneumoniae*, capable of intracellular proliferation, it does not enable the growth of extracellular pathogens like *E. coli*. In contrast to this, both pathogens similarly benefit from elevated iron concentration in an extracellular growth assay (**Supplementary Figure 3**).

Taken together, our experiments revealed that both, *E. coli* and *K. pneumoniae* can infect and invade AECII-derived A549 cells. Solely *K. pneumoniae* is capable of proliferating intracellularly, with increased growth capacity in iron-loaded cells.

### Alveolar Epithelial Cells Differentially Adapt Their Iron Metabolism to *E. coli* and *K. pneumoniae* Infection

**Figure 2:**
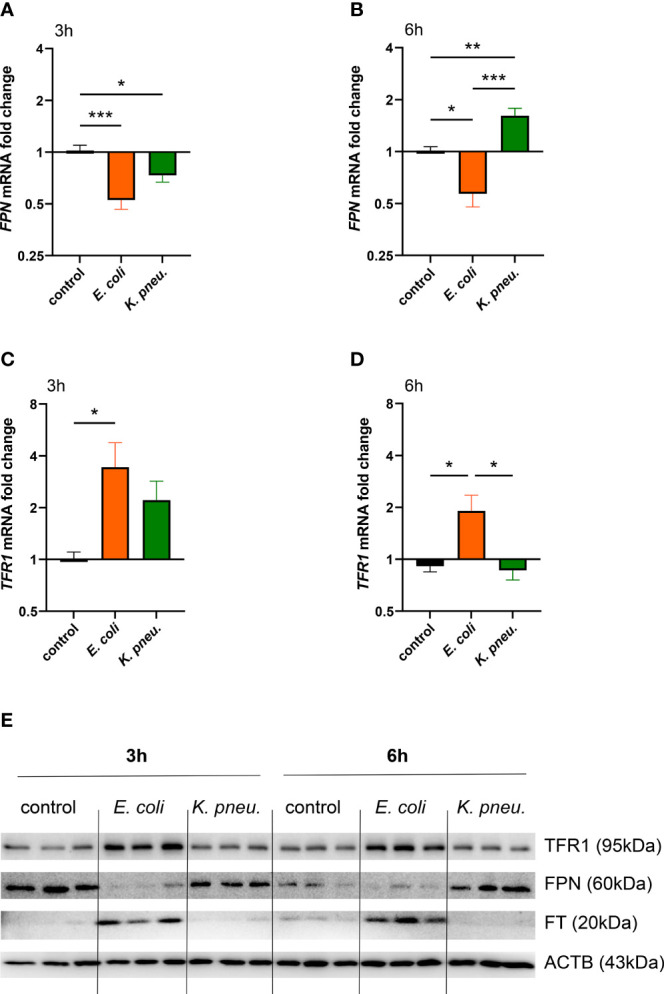
Differential expression of *FPN* **(A, B)** and *TFR1* **(C, D)** mRNA in infected A549 cells. A549 cells were infected for 2h with *E. coli* or *K. pneumoniae* at MOI of 10, subsequently washed thoroughly and harvested after 3h **(A–C)** or 6h **(B–D)** of intracellular infection. Data are shown as mean ± SEM of 3 separate experiments.Western blot of iron transport proteins in infected A549 cells **(E)**. A549 cells were infected for 2h with *E. coli* or *K. pneumoniae* at MOI of 10, subsequently washed thoroughly and harvested after 3h **(A, C)** or 6h **(B, D)** of intracellular infection. Representative image of three separate experiments.* denotes p<0.05, ** denotes p<0.01, *** denotes p<0.001 for *post-hoc* statistical testing. *K. pneu., K, pneumoniae*; TFR1, transferrin-receptor-1; FPN, ferroportin; FT, ferritin; ACTB, β-actin.

Next, we evaluated the differential gene and protein expression of key players of iron metabolism in A549 cells in response to infection with either pathogen. Three hours after the incubation phase, the only known ferrous iron exporter *FPN* showed decreased mRNA expression in cells infected with either pathogen (**Figure 2A**). Strikingly, after 6h of intracellular infection, *FPN* mRNA showed a differential regulation pattern in infected cells. While *E. coli* infected cells depicted persistent negative regulation, cells infected with *K. pneumoniae* revealed increased *FPN* mRNA levels (**Figure 2B**). At both time points, FPN protein levels (**Figure 2E** shows representative blot, **Supplementary Figures 4A**, **B** show densitometry of all blots) resembled this dynamic and pathogen-specific regulation pattern.

Notably, *TFR1* mRNA expression was increased solely in *E. coli* infected cells at both time points (**Figures 2C, D**) and TFR1 and FT protein levels were also drastically increased in cells challenged with the extracellular pathogen (**Figure 2E** and **Supplementary Figure 4**). In contrast to this, A549 cells infected with *K. pneumoniae* showed no upregulation of TFR1 and FT levels. This suggests that upon detection of intracellular bacterial proliferation, cellular iron sequestration is limited.

To summarize, the extracellular pathogen *E. coli* induces an iron retention phenotype, mainly characterized by the downregulation of the pivotal iron exporter FPN, the upregulation of the iron importer TFR1 and corresponding induction of the storage protein FT. Contrarily, cells infected with the intracellularly proliferating bacterium *K. pneumoniae* lacked induction of iron retention proteins and exhibited an increased iron export phenotype indicated by FPN upregulation.

### Pathogen-Specific Inflammatory Reaction of A549 Cells

**Figure 3:**
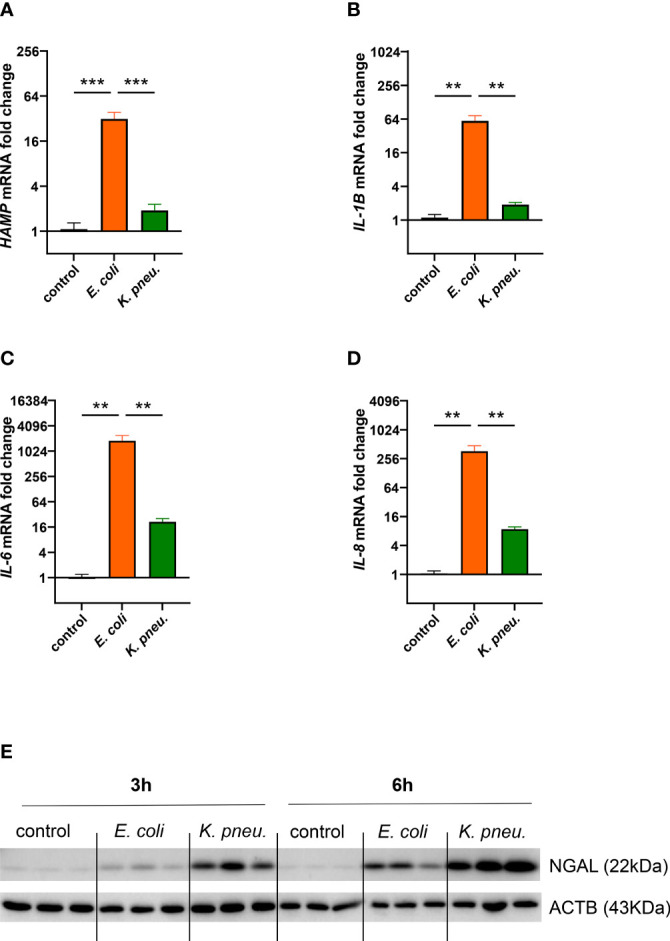
Differential mRNA expression of *HAMP* **(A)** and pro-inflammatory cytokines *IL-1B* **(B)** *IL-6* **(C)**, and *IL-8* **(D)** in infected A549 cells.A549 cells were infected for 2h with *E. coli* or *K. pneumoniae* at MOI of 10, subsequently washed thoroughly and harvested after 3h **(A)** or 6h **(B–D)** of intracellular infection. Data from three separate experiments are shown as mean ± SEM.Western blotting of NGAL in infected AECII **(E)**. A549 cells were infected for 2h with *E. coli* or *K. pneumoniae* at MOI of 10, subsequently washed thoroughly and harvested after 3h (left) or 6h (right) of intracellular infection. Representative image of three separate experiments. ** denotes p<0.01, *** denotes p<0.001 for *post-hoc* statistical testing. *K. pneu., K. pneumoniae*; HAMP, hepcidin antimicrobial peptide; NGAL, neutrophil gelatinase-associated lipocalin 2; ACTB, β-actin.

Next, we characterized the inflammatory reaction of AECII-derived A549 cells infected with either of the two pathogens. In our experiments, only cells infected with *E. coli* showed strong induction in *HAMP* mRNA, already after 3h of intracellular infection (**Figure 3A**). Conversely, these cells also depicted higher mRNA expression levels of pro-inflammatory cytokines *IL1B*, *IL-6* and *IL-8* at 6h of intracellular phase (**Figures 3B–D**).

Likewise, NGAL showed a differential regulation pattern in our infection model (**Figure 3E**). Moderate NGAL induction on the protein level was revealed in *E. coli* infected cells, most prominently at the 6h time point. In contrast, a steep increase of this siderophores-scavenger was induced in cells infected with *K. pneumoniae*.

Our experiments thus demonstrated pathogen-specific inflammatory reactions in infected epithelial cells. In *E. coli* infected cells, the expression of key iron regulator *HAMP*, as well as inflammatory cytokines were strongly induced. In contrast, the innate immune effector NGAL showed striking induction in *K. pneumoniae* infected cells, possibly related to the intracellular proliferation of this pathogen.

### Pathogen-Specific Reactions of A549 Cells Are Mediated by Oxidative Stress

**Figure 4:**
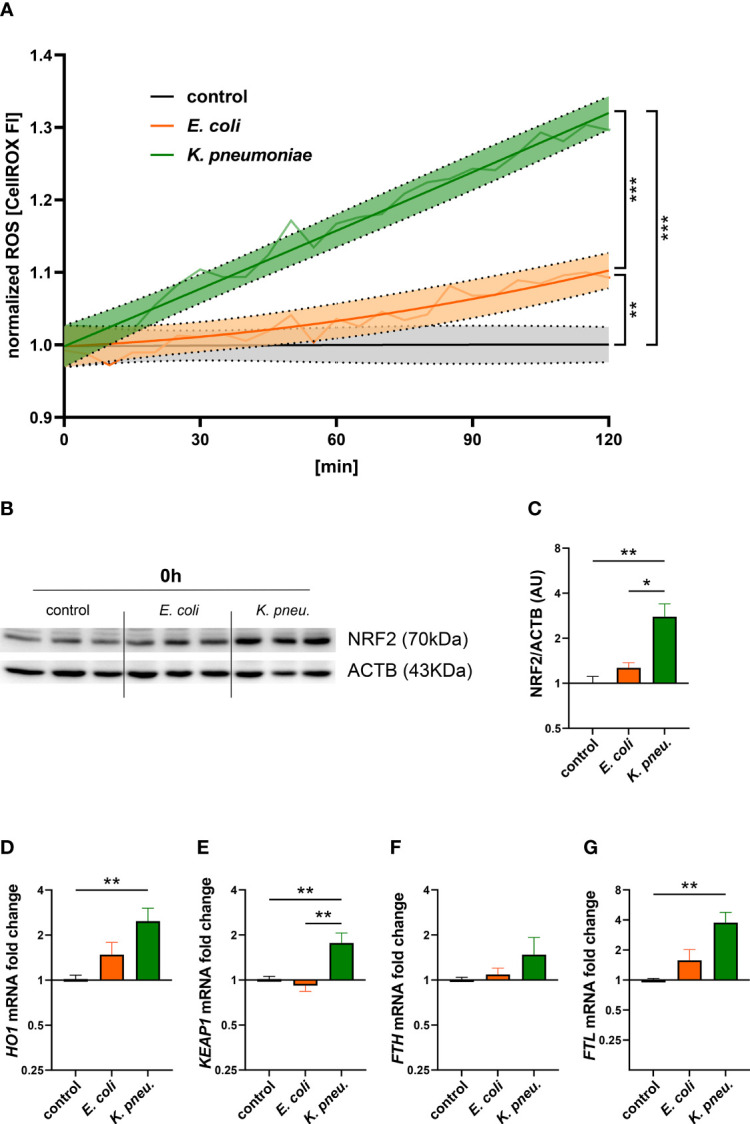
Time-course of ROS formation during the 2h incubation phase in A549 cells infected with either *E. coli* or *K. pneumoniae* **(A)**. The means of fluorescence intensity of three independent experiments are shown as a light colored line. Dark colored lines display a non-linear regression line-fit with 95% CI as error bands. Results were normalized to uninfected controls. Statistical testing was performed for the 120min time point. Western blot **(B)** of NRF2 and corresponding densitometry of two separate experiments **(C)** in infected A549 cells. A549 cells were infected for 2h with *E. coli* or *K. pneumoniae* at MOI of 10, subsequently washed thoroughly and directly harvested. Data are shown as mean ± SEM of 2 separate experiments. Differential mRNA expression of NRF2-targeted genes *HO1* **(D)**, *KEAP1* **(E)**, *FTH* **(F)**, and *FTL* **(G)** in infected A549 cells. A549 cells were infected for 2h with *E. coli* or *K. pneumoniae* at MOI of 10, subsequently washed thoroughly and harvested after 6h of intracellular infection. Data are shown as mean ± SEM of three separate experiments. * denotes p<0.05, ** denotes p<0.01, *** denotes p<0.001 for *post-hoc* statistical testing. ROS, reactive oxygen species; *K. pneu., K. pneumoniae*; NRF2, NF-E2-related factor 2; ACTB, β-actin; HO1, Heme oxygenase-1; KEAP1, Kelch-like ECH-associated protein 1; FTH, ferritin heavy chain; FTL, ferritin light chain.

Subsequently, we investigated possible mechanisms underlying pathogen-specific gene expression. To this end, we examined oxidative stress generation in A549 cells during the initial 2h incubation phase (**Figure 4A**). Our experiments showed significantly higher levels of reactive oxygen species (ROS) in cells infected with *K. pneumoniae*, compared to both, *E. coli* infected cells as well as uninfected controls. Elevated ROS levels, as found in *K. pneumoniae* infected cells, modulate cellular signaling and could thus at least partly explain the pathogen-specific reaction in iron homeostasis and inflammatory response (Spooner and Yilmaz, 2011). Correspondingly, Western blots of nuclear extracts revealed higher levels of the oxidative-stress-response transcription factor NF-E2-related factor 2 (NRF2) in cells infected with *K. pneumoniae* as compared to *E. coli* infected cells and uninfected controls (**Figures 4B, C**).

Furthermore, we analyzed differential gene expression of NRF2 pathway-related- as well as target-genes involved in iron metabolism. Appropriately, *Kelch-like ECH-associated protein 1* (*KEAP1*) mRNA expression was increased in *K. pneumoniae* infected A549 cells (**Figure 4E**). *Heme oxygenase-1* (*HO1*), a phase II detoxifying enzyme and NRF2 target gene, showed significant mRNA upregulation in cells infected with *K. pneumoniae* (**Figure 4D**). Another target gene is the iron storage protein FT (Cairo et al., 1995). After analyzing both, *ferritin heavy*- (*FTH*) and *light*- (*FTL*) chain transcripts, we found a significant increase of only *FTL* mRNA levels in *K. pneumoniae* infected cells (**Figures 4F, G**). In addition, treatment with the ROS-scavenger NAC revealed a negative effect on the expression of NRF2- related genes in *K. pneumoniae* infected cells (**Supplementary Figure 5**).

## Discussion

**Figure 5:**
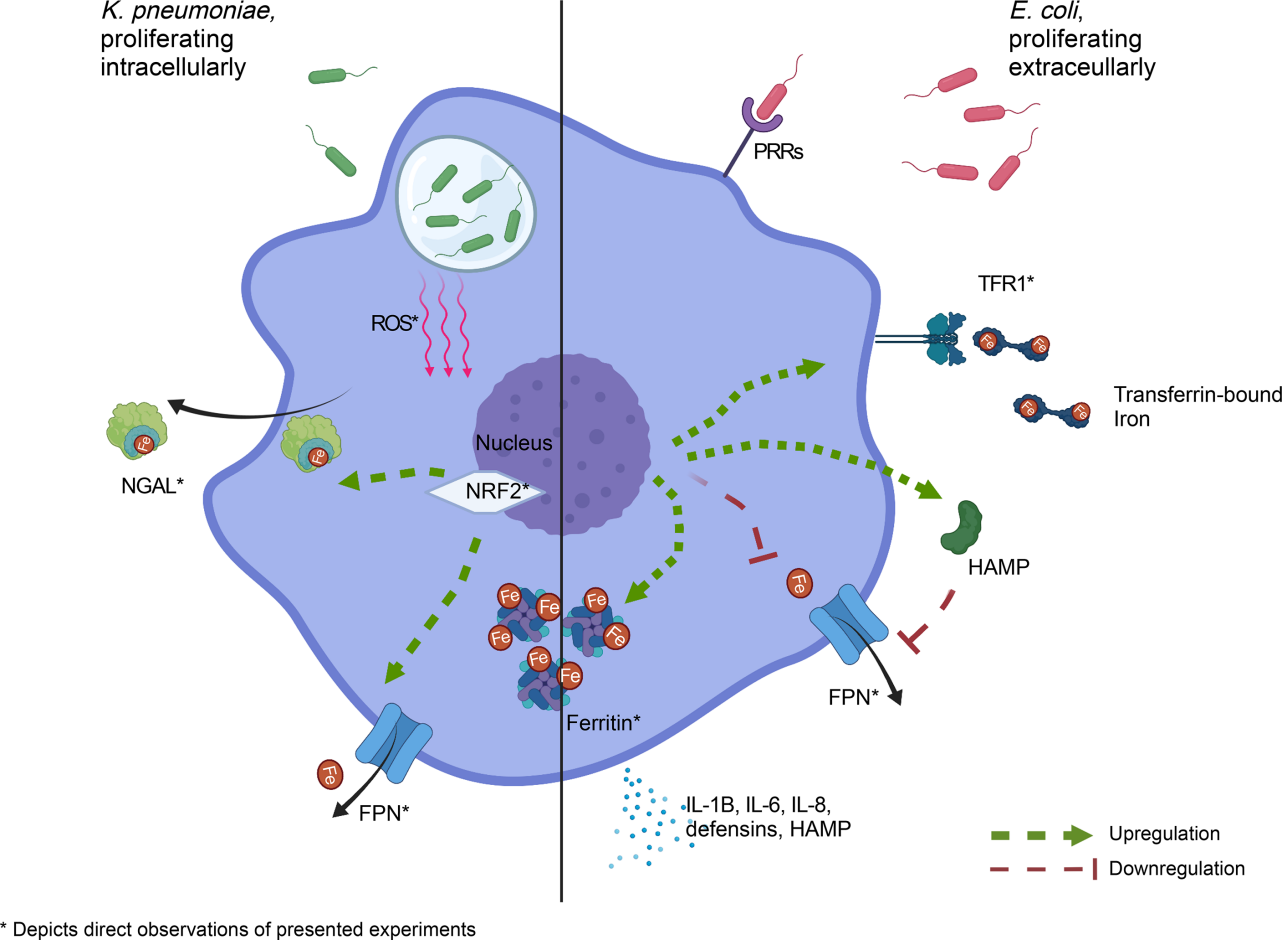
Pathogen-specific adaption of cellular iron homeostasis in AECII. Left panel: *K. pneumoniae*, capable of proliferating intracellularly, elicits an iron-export phenotype, likely mediated by oxidative stress. Right panel: *E. coli*, which proliferates predominantly in the extracellular space, induces an iron retention phenotype in infected AECII. * denotes direct experimental observations AECII, type-2 alveolar epithelial cell; ROS, reactive oxygen species; NGAL, neutrophil gelatinase-associated lipocalin 2; PRR, pattern recognition receptor; TFR1, transferrin receptor-1; FPN, ferroportin; NRF2, NF-E2-related factor 2; Created with BioRender.com.

AECII are amongst the first cell types to encounter respiratory pathogens. Unlike alveolar macrophages, AECII are not typically considered to be ´professional´ immune cells, but still express various pattern-recognition receptors (PRRs) (Bals and Hiemstra, 2004). In this study, we provide first *in-vitro* evidence that epithelial cells are able to mount a pathogen-specific nutritional immune response. Specifically, A549 cells infected with *K. pneumoniae* which resides intracellularly (de Astorza et al., 2004; Hsu et al., 2015) induce the expression of the iron exporter FPN. In contrast, A549 cells display an iron-retention phenotype when exposed to *E. coli*, a prototypical extracellular bacterium. This ability to differentially regulate cellular iron metabolism in response to infection with intra- or extracellular bacteria has hitherto only been ascribed to myeloid cells. A proposed model of this pathogen-specific reaction of AECII to invading bacteria is depicted in **Figure 5**.

We found higher bacterial loads in iron-loaded cells infected with *K. pneumoniae*, emphasizing this metal’s crucial role in infection. Supporting these findings, higher available iron in the pathogen’s compartment is associated with increased bacterial growth in various models (Pan et al., 2010; Schmidt et al., 2018; Haschka et al., 2021b; Hoffmann et al., 2021). Thus, it is pivotal for the host to deprive iron of an invading pathogen during the spatio-temporal dynamics of the host-pathogen interaction. Specifically, the balance between iron uptake, export and storage needs to be adapted to the niche of the invading pathogen. This raises the question of how the pathogen is sensed and how this information is subsequently translated into altered cellular iron handling.

Typically, downstream signaling of PRRs such as toll-like-receptors (TLRs) is responsible for early innate immune responses to bacteria and bacterial components (Knapp et al., 2008). Specifically, TLR-4 mediated detection of bacterial lipopolysaccharide (LPS) showed to be responsible for inflammatory cytokine induction in airway epithelial cells, an effect that could be diminished by TLR4-blockade (Grandel et al., 2009; John et al., 2010). In the case of iron homeostasis, the expression of the only known ferrous iron exporter FPN is transcriptionally repressed by TLR signaling (Ludwiczek et al., 2003). This mechanism is likely mediating the transcriptional response in infected A549 cells, as we found decreased *FPN* mRNA expression in infected cells with both pathogens in the early phase of infection. Strikingly, we found *FPN* mRNA levels increased in cells infected with intracellularly proliferating *K. pneumoniae* at the later time interval, possibly decreasing iron availability to the pathogen. This positive *FPN* regulation pattern seems to be dependent on the metabolism and/or proliferation of a viable intracellular pathogen, as revealed by experiments with heat-killed bacteria: The challenge of cells with heat-killed bacteria led to a negative *FPN* regulation pattern at early and late time points, possibly because of the high abundance of pathogen-associated molecules, derived from either bacterium (**Supplementary Figure 6**). In myeloid cells infected with intracellularly proliferating *Salmonella*, a very similar dynamic regulation of FPN expression has been observed (Nairz et al., 2007). In contrast, cells infected with the predominantly extracellular pathogen *E. coli* revealed an iron-retention-phenotype, depicted by TFR1 and FT upregulation. This finding is parallel to several cell types reacting to extracellular bacteria with iron sequestration, to withhold this essential nutrient from invading pathogens (Healy et al., 2021).

At the interface of iron and immunity, the hepcidin antimicrobial peptide (HAMP) acts as a key regulator of systemic (endocrine) and localized (paracrine) iron metabolism (Theurl et al., 2008). HAMP binds the iron exporter FPN, leading to its internalization and degradation, thus increasing intracellular iron sequestration (Nemeth et al., 2004). AECII also express HAMP, suggested to have a paracrine function in the lung (Chen et al., 2014; Zhang et al., 2019). Upon systemic infection, inflammatory cytokines like IL-6 lead to increased *HAMP* transcription *via* signal transducer and activator of transcription (STAT)-3 in hepatocytes (Wrighting and Andrews, 2006). Intriguingly, *HAMP* induction has also been reported by LPS treatment independent of IL-6 in various cell types, including alveolar macrophages (Nguyen et al., 2006; Lee et al., 2017). In the local environment of infection, direct sensing of bacterial components could thus affect cellular iron transport. Our experiments showed that both, inflammatory cytokines as well as *HAMP* expression were significantly increased in *E. coli* infected A549 cells, but HAMP could not be detected in supernatants of infected cells *via* ELISA (data not shown).

Our data thus suggests that the iron sequestration phenotype depicted by *E. coli* infected cells is accomplished *via* intracellular signaling following pathogen sensing, a response very similar to the response observed in alveolar macrophages (Nguyen et al., 2006).

It is of note, that adaption of iron metabolism in a state of inflammation not only affects the microenvironment of infection but may also disrupt systemic iron allocation. Increased iron retention, caused by chronic state of inflammation in infections, proliferative or autoimmune disorders may render iron inaccessible for erythropoiesis, leading to anemia of chronic disease (Weiss and Goodnough, 2005; Valente de Souza et al., 2021).

Interestingly, upon infection, various bactericidal mechanisms have been described in AECII. One such anti-microbial immune effector molecule produced by AECII in response to bacterial infection is NGAL (Saiga et al., 2008; Ruaro et al., 2021). NGAL takes part in inhibiting bacterial iron acquisition by binding bacterial siderophores and plays a significant role in the lung’s innate immune defense against *Enterobacteriacae* (Chan et al., 2009; Wu et al., 2010). Our experiments revealed a differential regulation pattern of this immune molecule in A549 cells, with only moderate induction in *E. coli* infected cells, compared to striking induction in *K. pneumoniae* infected cells. Moderate induction could be explained by autocrine effects of inflammatory cytokines, like IL-1beta, as sole bacterial LPS stimulation does not translate into increased NGAL expression (Cowland et al., 2003). In contrast, high NGAL induction in the lung has been reported with other intracellular bacteria (Guglani et al., 2012). As another factor possibly at play, *K. pneumoniae* is capable of NGAL evasion by producing alternative siderophores, which the immune molecule cannot bind, possibly translating into a bacterial survival advantage (Bachman et al., 2012). At last, NGAL overexpression is also linked to oxidative stress, as elevated cellular levels of ROS lead to NGAL induction in myeloid cells (Fritsche et al., 2012). Furthermore, NGAL itself exerts antioxidant effects, thus ameliorating ROS-mediated toxicity (Roudkenar et al., 2011). Accordingly, we found elevated ROS levels in *K. pneumoniae* infected A549 cells. This finding is in line with intracellular pathogens being associated with ROS-generation in host cells, and with reports of *K. pneumoniae* infected airway epithelial cells suffering from increased oxidative stress (Shin et al., 2010; Spooner and Yilmaz, 2011; Leone et al., 2016). Of note, NGAL can export iron out of macrophages by employing a mammalian siderophore and thereby limiting access of iron for bacteria residing within macrophages (Nairz et al., 2009). However, this may be of benefit for some bacteria, such as *Streptococcus pneumoniae*, were NGAL expression was associated with an impaired infection control (Warszawska et al., 2013).

Pathogen-specific reactions regarding the inflammatory response and iron homeostasis could at least partly be explained by differences in elicited cellular ROS. After detecting oxidative stress, the transcription factor NRF2 initiates a whole cassette of cytoprotective genes including iron transporters and has also been linked to host defense against bacteria (Battino et al., 2018). NRF2 controls its own degradation through an auto-regulatory negative feedback loop. Its activation leads to increased expression of *KEAP1*, the primary inhibitor of NRF2 (Lee et al., 2007). Consequently, our experiments revealed higher protein levels of NRF2 as well as target gene induction (*FPN*, *FTL*, *HO1*, *KEAP1*) in *K. pneumoniae* infected cells. In line with these findings, NRF2 target genes showed a trend towards reduced expression in *K. pneumoniae* infected cells, when treated with the ROS-scavenger NAC. Of note, cellular protein concentrations of the NRF2-target FT are primarily regulated post-transcriptionally (Torti and Torti, 2002), explaining the discrepancy between mRNA regulation and total cellular protein abundance. In macrophages infected with intracellular bacteria, the pivotal upregulation of FPN was shown to be NRF2 dependent (Nairz et al., 2013). Underlining this relationship, a mouse pneumonia model showed NRF2 null animals infected with *Streptococcus pneumoniae* suffered from increased mortality rates (Gomez et al., 2016).

To conclude, we could demonstrate pathogen-specific inflammatory and innate functions in AECII-derived A549 cells, which are in line with the concept of *nutritional immunity*. Multi-drug resistant bacteria, including carbapenem-resistant *K. pneumoniae* and extended-spectrum beta-lactamase-producing *E. coli*, are continually emerging and are a critical priority global health concern (Pitout et al., 2015). An improved understanding of innate and adaptive immune functions, including *nutritional immunity*, might provide new treatment options for patients infected with these multi-drug resistant bacteria.

## Data Availability Statement

The raw data supporting the conclusions of this article will be made available by the authors, without undue reservation.

## Author Contributions

MN, GW, and IT planned and designed the project. PG, AH, RH, and MS performed experiments. PG did the visualization of the data and performed the statistical analysis. PG and MN prepared and created the initial draft. AH, IT, GW, TS, NB, and RH were included in the critical review and writing of the manuscript. MN, IT, and GW were responsible for supervision and funding acquisition. All authors contributed to the article and approved the submitted version.

## Funding

This project was enabled and supported by grants of the Austrian Science Fund (FWF, DOC 82 doc.fund; doctoral program MCBD).

## Conflict of Interest

The authors declare that the research was conducted in the absence of any commercial or financial relationships that could be construed as a potential conflict of interest.

## Publisher’s Note

All claims expressed in this article are solely those of the authors and do not necessarily represent those of their affiliated organizations, or those of the publisher, the editors and the reviewers. Any product that may be evaluated in this article, or claim that may be made by its manufacturer, is not guaranteed or endorsed by the publisher.

## Supplementary Material

Supplementary Figure 1 Timeline of the experimental setup. After a 2h incubation phase with either model pathogen, cells were washed and incubated in a gentamicin-containing medium for the intracellular infection phase. Cells were harvested at different time points for analysis, as depicted.

Supplementary Figure 2 Additional immune fluorescence images reveal the predominant localization of bacteria at lower magnification: *E. coli* in the extracellular space **(A)** and *K. pneumoniae* in the intracellular space **(B)** of A549 cells. Images show Ypet expressing bacteria (green) infecting A549 cells (DAPI= blue, phalloidin= yellow) at 200x magnification with a 50µm scale bar.

Supplementary Figure 3 Growth assay of *E. coli* **(A)** or *K. pneumoniae* **(B)**. Bacteria in logarithmic growth phase were diluted to an OD**_600_** 0.005 in cell culture medium or medium supplemented with 25µM iron (III) nitrate nonahydrate. OD**_600_** was continually measured over 600 minutes, means ± 95% CI (n=5) are shown.

Supplementary Figure 4 Densitometry of Western blots of key iron metabolism proteins FPN **(A, B)**, TFR1 **(C, D)**, FT **(E, F)** and NGAL **(G, H)** in infected A549 cells. A549 cells were infected for 2h with *E. coli* or *K. pneumoniae* at MOI of 10, subsequently washed thoroughly and harvested after 3h (left) or 6h (right) of intracellular infection. Data from three separate experiments are shown as mean ± SEM. * denotes p<0.05, ** denotes p<0.01, *** denotes p<0.001 for *post-hoc* statistical testing. *K. pneu.*, *K. pneumoniae*; FPN, ferroportin; TFR1, transferrin-receptor-1; FT, ferritin; NGAL, neutrophil gelatinase-associated lipocalin 2.

Supplementary Figure 5 Differential mRNA expression of NRF2-associated genes *FPN* **(A)**, *HO1* **(B)**, *FTH* **(C)**, *FTL* **(D)**. and *KEAP1* **(E)** in infected A549 cells, treated with ROS-scavenger NAC. A549 cells were infected for 2h with *E. coli* or *K. pneumoniae* at MOI of 10, subsequently washed thoroughly and harvested after 6h of intracellular infection. Cells were treated with 5mM NAC 20 min before infection, and during intracellular infection. Data (n=3) shown as mean ± SEM, normalized to corresponding solvent controls ROS, reactive oxygen species; NAC, N-acetyl-cysteine; *K. pneu*., *K. pneumoniae*; NRF2, NF-E2-related factor 2; *FPN*, ferroportin; HO1, Heme oxygenase-1; FTH, ferritin heavy chain; FTL, ferritin light chain; KEAP1, Kelch-like ECH-associated protein 1.

Supplementary Figure 6 Differential mRNA expression of *FPN* in cells treated with heat-killed bacteria. A549 cells were treated with heat-killed (HK) *E. coli* or *K. pneumoniae* at MOI of 100 for 2h, subsequently washed thoroughly and harvested after 3h **(A)** and 6h **(B)**. Data (n=3) shown as mean ± SEM, normalized to untreated controls. HK, heat-killed; *K. pneu*., *K. pneumoniae*; *FPN*, ferroportin.

## Abbreviations

µg, microgram; µM, micromolar; µm, micrometer; ACTB, actin beta; AECII, type-2 alveolar epithelial cells; CFU, colony forming units; *E. coli*, Escherichia coli; Fig., figure; FPN, ferroportin; FT, ferritin; FTH, ferritin heavy chain; FTL, ferritin light chain; h, hour; HAMP, hepcidin antimicrobial peptide; HO1, heme oxygenase-1; IL-1B, interleukin 1 beta; IL-6, interleukin 6; IL-8, interleukin 8; *K. pneu., Klebsiella pneumonia;* LPS, lipopolysaccharide; min, minute; KEAP1, Kelch-like ECH-associated protein 1; NAC, N-acetyl-cysteine; NGAL, Neutrophil gelatinase-associated lipocalin 2;NRF2, NF-E2-related factor 2; OD**_600_**, optical density (600nm); Qpcr, quantitative real-time polymerase chain reaction; ROS, reactive oxygen species; STAT3, signal transducer and activator of transcription – 3; TFR1, transferrin-receptor-1; TLR4, toll-like receptor 4.
