# Supplementary figures and images for: Airway Epithelial Cells Differentially Adapt Their Iron Metabolism to Infection With Klebsiella pneumoniae and Escherichia coli In Vitro
Source: Front Cell Infect Microbiol. 2022 May 18;12:875543. doi: 10.3389/fcimb.2022.875543 (PMC9157649; doi:10.3389/fcimb.2022.875543)

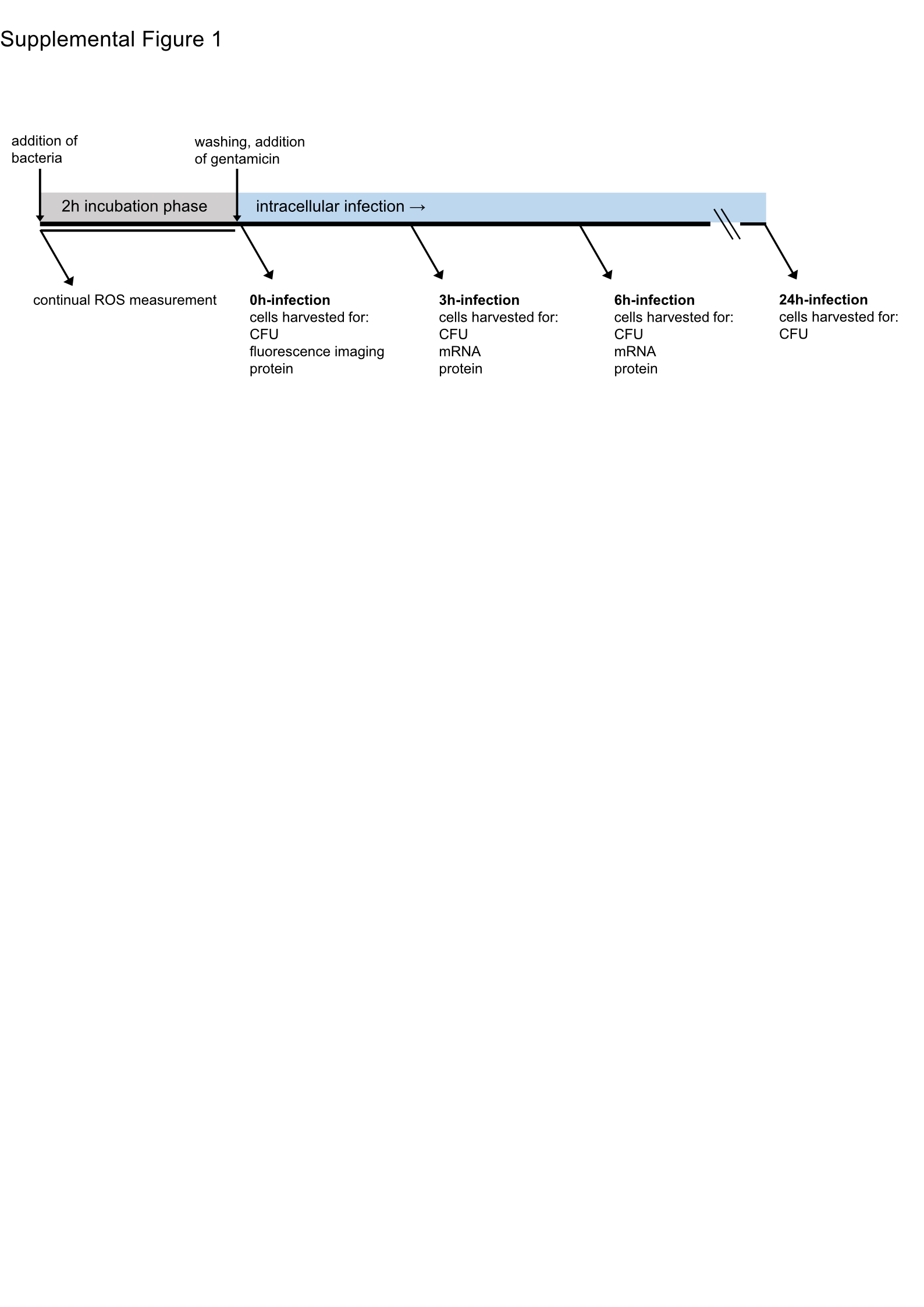

Supplement: Supplementary Figure 1 — Timeline of the experimental setup. After a 2h incubation phase with either model pathogen, cells were washed and incubated in a gentamicin-containing medium for the intracellular infection phase. Cells were harvested at different time points for analysis, as depicted. [file Image_1.tiff]

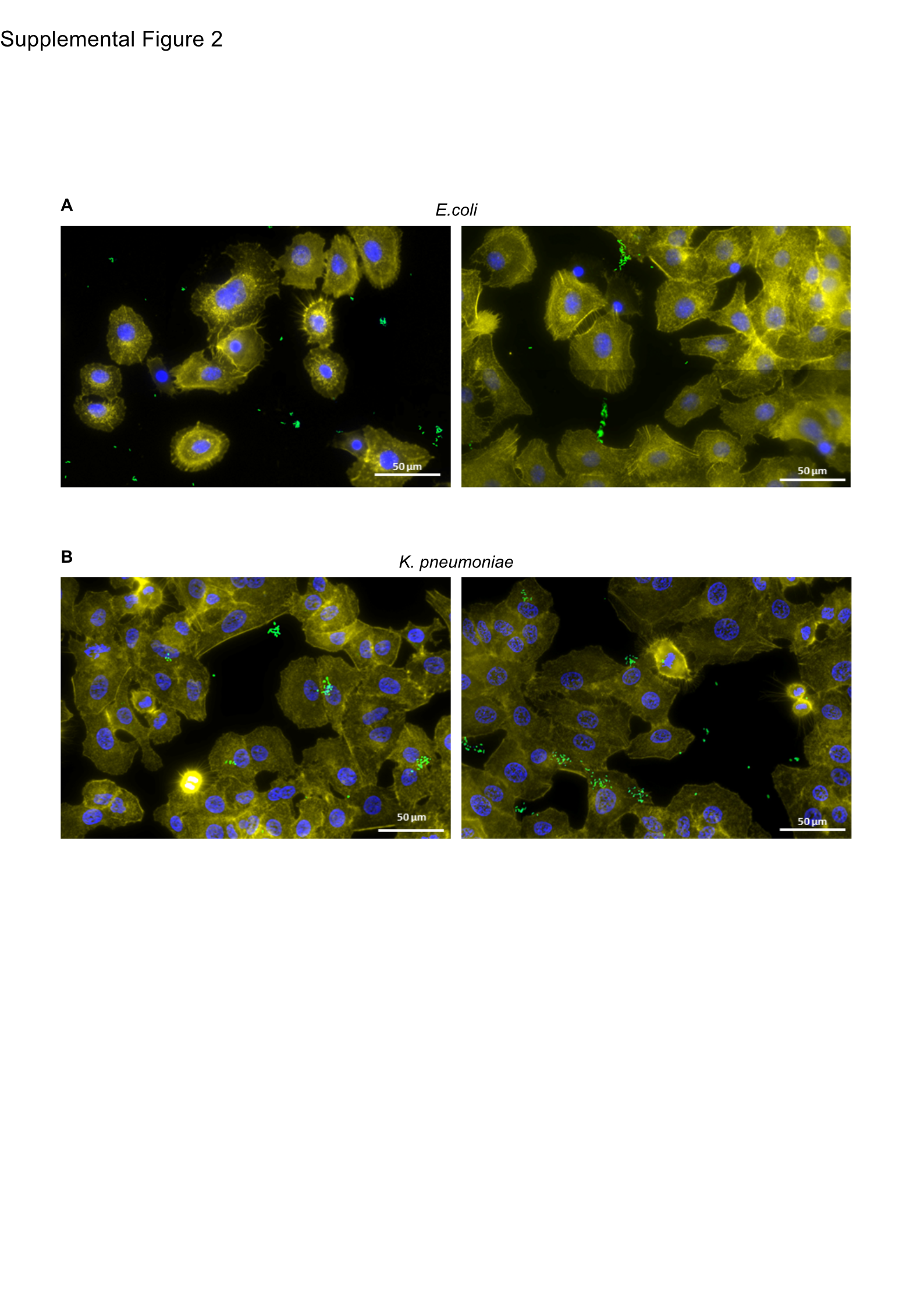

Supplement: Supplementary Figure 2 — Additional immune fluorescence images reveal the predominant localization of bacteria at lower magnification: E. coli in the extracellular space (A) and K. pneumoniae in the intracellular space (B) of A549 cells. Images show Ypet expressing bacteria (green) infecting A549 cells (DAPI= blue, phalloidin= yellow) at 200x magnification with a 50µm scale bar. [file Image_2.tiff]

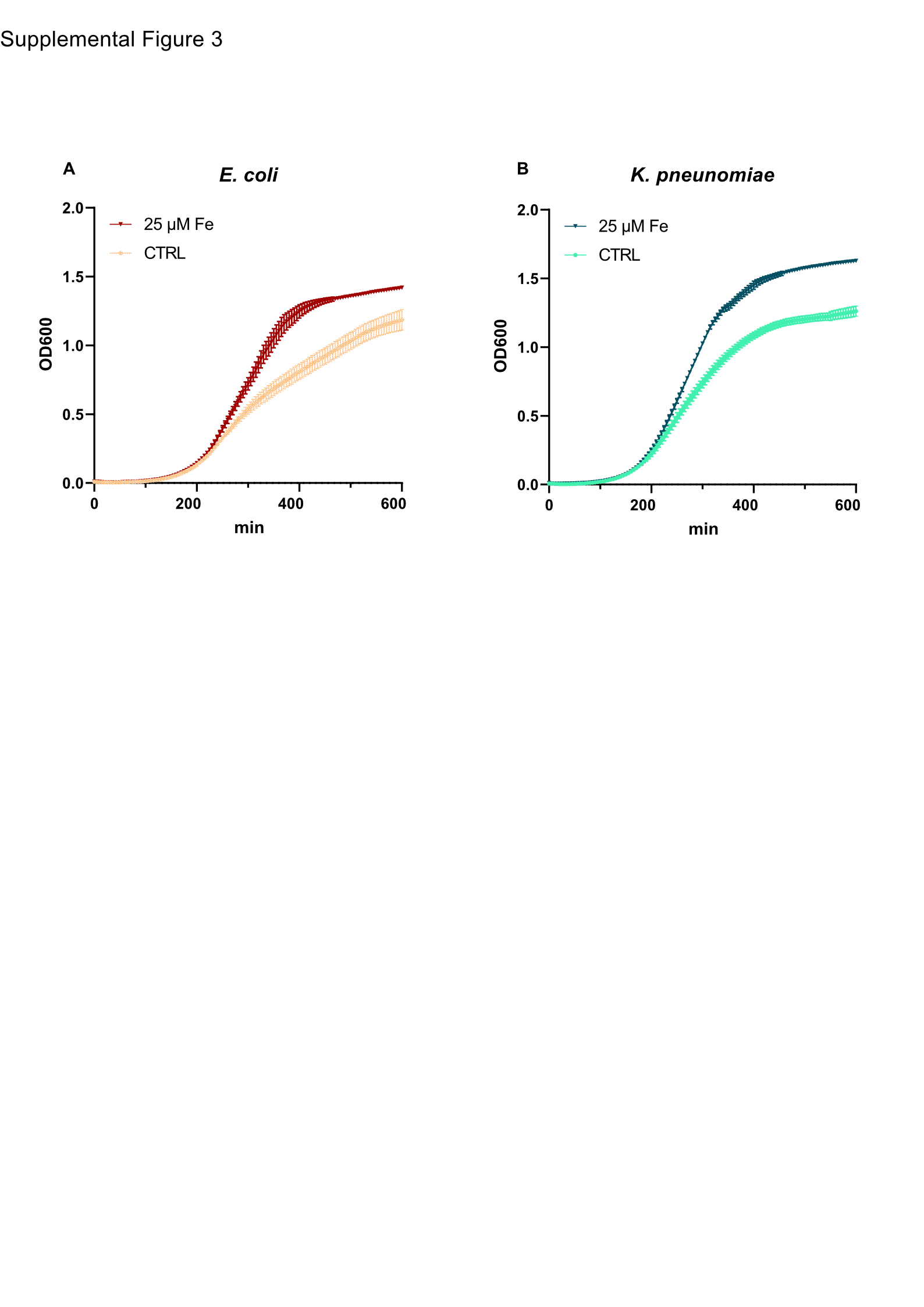

Supplement: Supplementary Figure 3 — Growth assay of E. coli (A) or K. pneumoniae (B). Bacteria in logarithmic growth phase were diluted to an OD 600 0.005 in cell culture medium or medium supplemented with 25µM iron (III) nitrate nonahydrate. OD 600 was continually measured over 600 minutes, means ± 95% CI (n=5) are shown. [file Image_3.tiff]

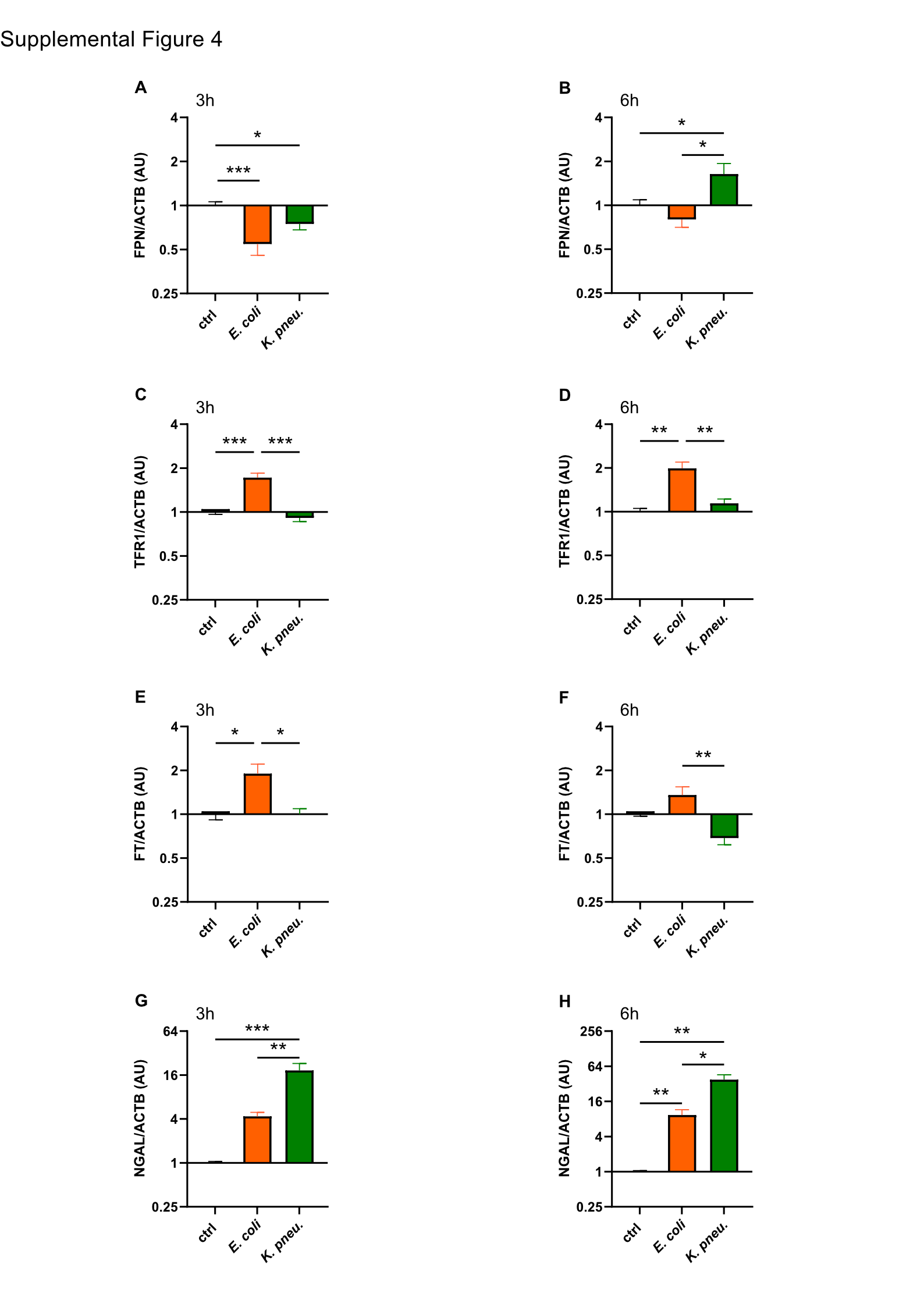

Supplement: Supplementary Figure 4 — Densitometry of Western blots of key iron metabolism proteins FPN (A, B), TFR1 (C, D), FT (E, F) and NGAL (G, H) in infected A549 cells. A549 cells were infected for 2h with E. coli or K. pneumoniae at MOI of 10, subsequently washed thoroughly and harvested after 3h (left) or 6h (right) of intracellular infection. Data from three separate experiments are shown as mean ± SEM. * denotes p<0.05, ** denotes p<0.01, *** denotes p<0.001 for post-hoc statistical testing. K. pneu., K. pneumoniae; FPN, ferroportin; TFR1, transferrin-receptor-1; FT, ferritin; NGAL, neutrophil gelatinase-associated lipocalin 2. [file Image_4.tiff]

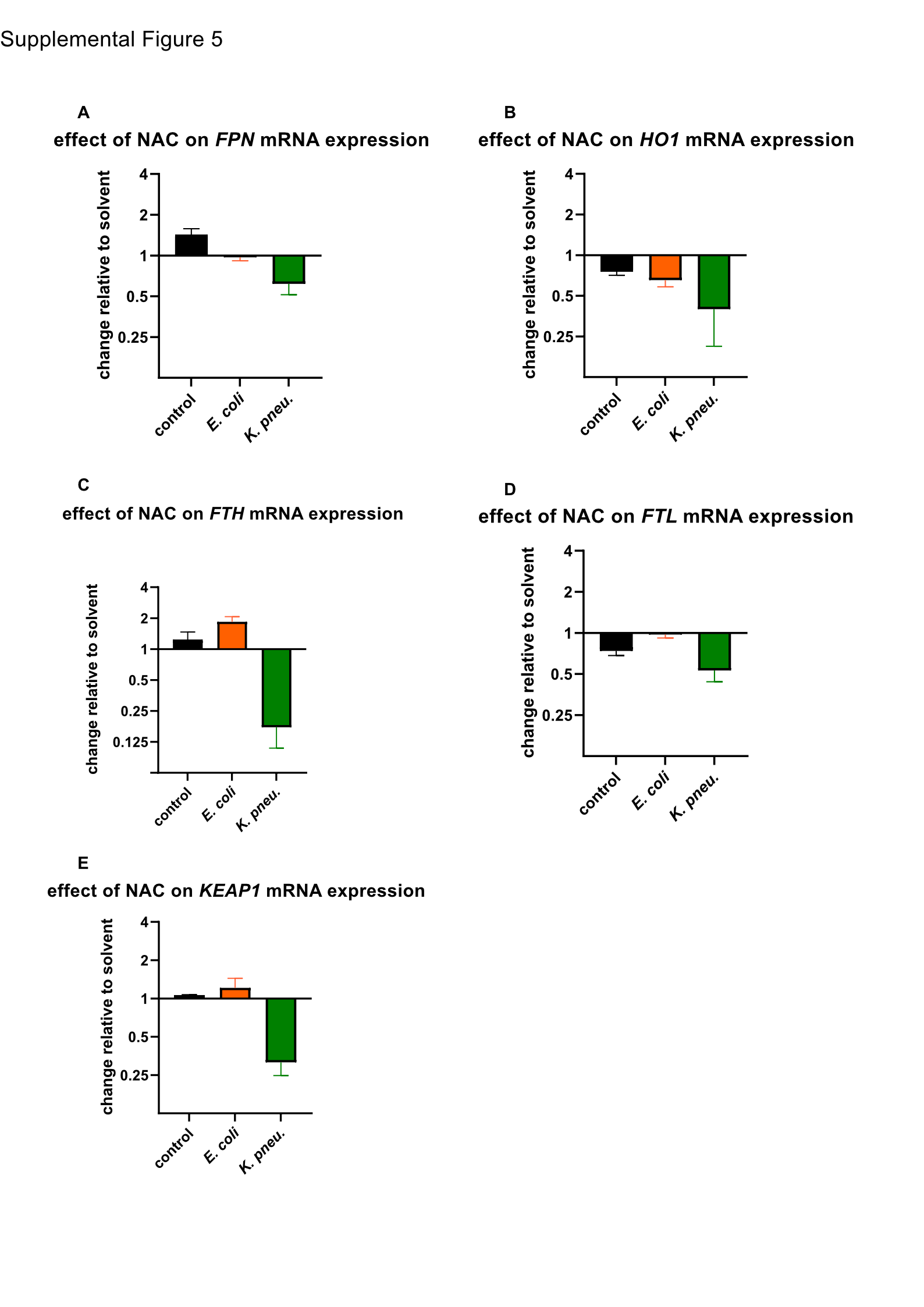

Supplement: Supplementary Figure 5 — Differential mRNA expression of NRF2-associated genes FPN (A), HO1 (B), FTH (C), FTL (D). and KEAP1 (E) in infected A549 cells, treated with ROS-scavenger NAC. A549 cells were infected for 2h with E. coli or K. pneumoniae at MOI of 10, subsequently washed thoroughly and harvested after 6h of intracellular infection. Cells were treated with 5mM NAC 20 min before infection, and during intracellular infection. Data (n=3) shown as mean ± SEM, normalized to corresponding solvent controls ROS, reactive oxygen species; NAC, N-acetyl-cysteine; K. pneu., K. pneumoniae; NRF2, NF-E2-related factor 2; FPN, ferroportin; HO1, Heme oxygenase-1; FTH, ferritin heavy chain; FTL, ferritin light chain; KEAP1, Kelch-like ECH-associated protein 1. [file Image_5.tiff]

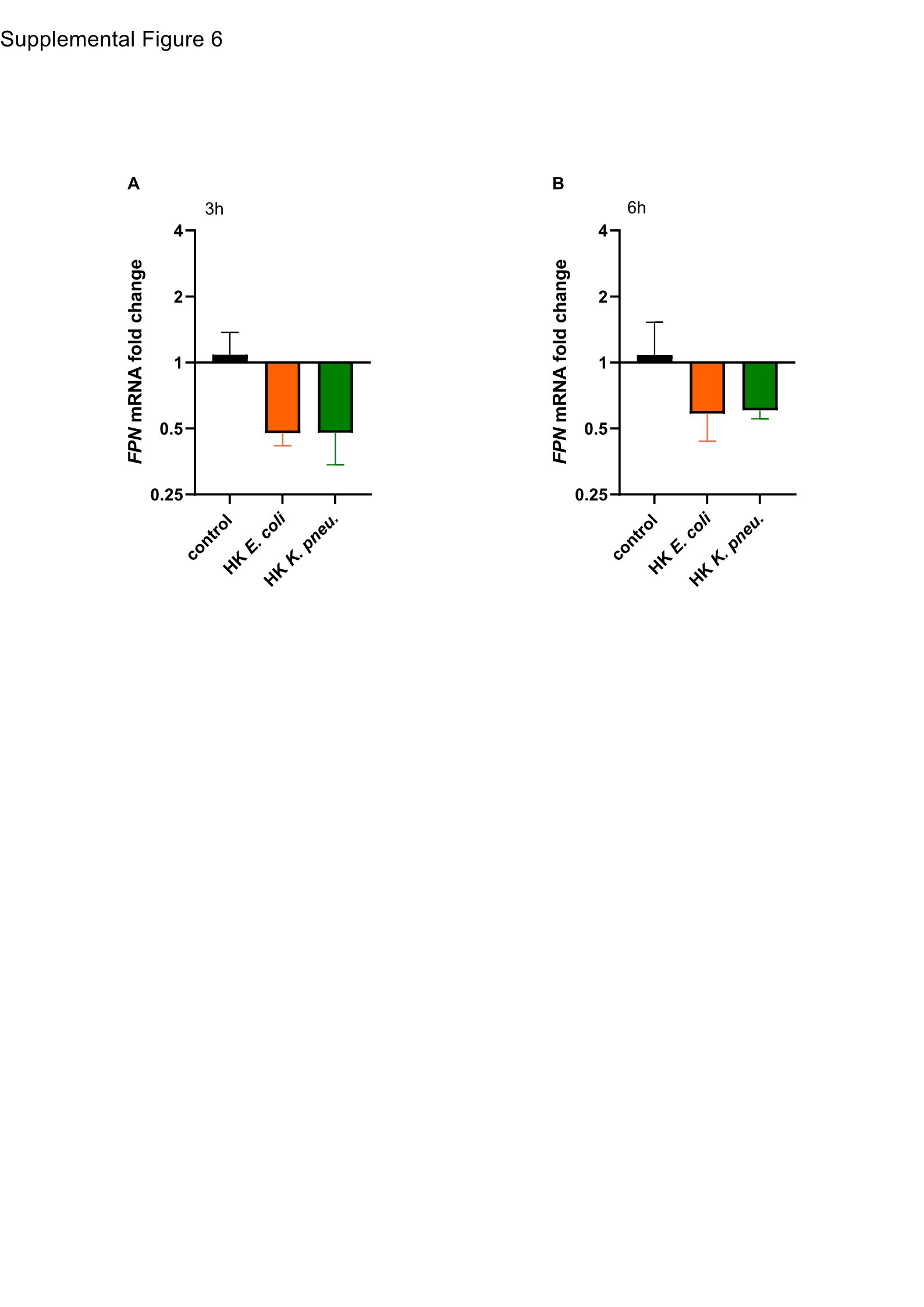

Supplement: Supplementary Figure 6 — Differential mRNA expression of FPN in cells treated with heat-killed bacteria. A549 cells were treated with heat-killed (HK) E. coli or K. pneumoniae at MOI of 100 for 2h, subsequently washed thoroughly and harvested after 3h (A) and 6h (B). Data (n=3) shown as mean ± SEM, normalized to untreated controls. HK, heat-killed; K. pneu., K. pneumoniae; FPN, ferroportin. [file Image_6.tiff]
